# Androgen receptor protein is down-regulated by basic fibroblast growth factor in prostate cancer cells

**DOI:** 10.1054/bjoc.1999.0874

**Published:** 1999-12-08

**Authors:** M V Cronauer, C Nessler-Menardi, H Klocker, K Maly, A Hobisch, G Bartsch, Z Culig

**Affiliations:** Departments of Urology, Medical Chemistry and Biochemistry, University of Innsbruck, Anichstraße 35, Innsbruck, A-6020, Austria; Department of Urology, Northwestern University Medical School, Robert H Lurie Comprehensive Cancer Center – Olson 8360, 710 N Fairbanks Court Chicago IL 6061, USA

**Keywords:** basic fibroblast growth factor, androgen receptor, prostate cancer, calcium influx

## Abstract

Interactions between polypeptide growth factors and the androgen receptor (AR) are important for regulation of cellular events in carcinoma of the prostate. Basic fibroblast growth factor (bFGF), the prototype of heparin-binding growth factors, and the AR are commonly expressed in prostate cancer. bFGF diminished prostate-specific antigen protein in the supernatants of androgen-stimulated human prostate cancer cells LNCaP by 80%. In the present study, we asked whether the bFGF effect on prostate-specific antigen is preceded by action on AR expression. LNCaP cells were treated with bFGF and AR protein expression was determined by immunoblotting and ligand binding assay. bFGF down-regulated AR protein in a dose-dependent manner showing a maximal effect at 50 ng ml^−1^both in the presence or absence of dihydrotestosterone. Down-regulation of AR protein expression occurred already after 8 h of bFGF treatment and a maximal inhibition was observed 24 h after addition of bFGF to culture media. As AR expression can be reduced by an increase in intracellular calcium levels, we investigated whether the bFGF effect on AR protein is mediated by this mechanism. Calcium release from intracellular stores and store-operated calcium influx after treatment with either bFGF or calcium ionophore A 23187 were measured by single cell fluorescence technique. The ionophore A 23187 was able to induce calcium influx and an increase in cytoplasmic calcium concentration in LNCaP cells. In contrast, bFGF was incapable of eliciting a similar effect. In contrast to AR protein, AR mRNA levels were not affected by bFGF as shown by semiquantitative reverse transcription polymerase chain reaction. In summary, these studies show that bFGF is a potent negative regulator of AR protein expression in the human prostate cancer cell line LNCaP. © 2000 Cancer Research Campaign


*British Journal of Cancer* (2000) **82**(1), 39–45
© 2000 Cancer Research Campaign
Article no. bjoc.1999.0874

### Androgen receptor protein is down-regulated by basic fibroblast growth factor in prostate cancer cells

**MV Cronauer[1,3], C Nessler-Menardi[1], H Klocker[1], K Maly[2], A Hobisch[1], G Bartsch[1] and Z Culig[1]**

Departments of [1]Urology and [2]Medical Chemistry and Biochemistry, University of Innsbruck, Anichstraße 35, A-6020 Innsbruck, Austria; [3] Department of Urology, Northwestern University Medical School, Robert H Lurie Comprehensive Cancer Center – Olson 8360, 710 N Fairbanks Court, Chicago IL 6061, USA

**Summary** Interactions between polypeptide growth factors and the androgen receptor (AR) are important for regulation of cellular events in carcinoma of the prostate. Basic fibroblast growth factor (bFGF), the prototype of heparin-binding growth factors, and the AR are commonly expressed in prostate cancer. bFGF diminished prostate-specific antigen protein in the supernatants of androgen-stimulated human prostate cancer cells LNCaP by 80%. In the present study, we asked whether the bFGF effect on prostate-specific antigen is preceded by action on AR expression. LNCaP cells were treated with bFGF and AR protein expression was determined by immunoblotting and ligand binding assay. bFGF down-regulated AR protein in a dose-dependent manner showing a maximal effect at 50 ng $ml^{-1}$ both in the presence or absence of dihydrotestosterone. Down-regulation of AR protein expression occurred already after 8 h of bFGF treatment and a maximal inhibition was observed 24 h after addition of bFGF to culture media. As AR expression can be reduced by an increase in intracellular calcium levels, we investigated whether the bFGF effect on AR protein is mediated by this mechanism. Calcium release from intracellular stores and store-operated calcium influx after treatment with either bFGF or calcium ionophore A 23187 were measured by single cell fluorescence technique. The ionophore A 23187 was able to induce calcium influx and an increase in cytoplasmic calcium concentration in LNCaP cells. In contrast, bFGF was incapable of eliciting a similar effect. In contrast to AR protein, AR mRNA levels were not affected by bFGF as shown by semiquantitative reverse transcription polymerase chain reaction. In summary, these studies show that bFGF is a potent negative regulator of AR protein expression in the human prostate cancer cell line LNCaP. © 2000 Cancer Research Campaign

**Keywords:** basic fibroblast growth factor; androgen receptor; prostate cancer; calcium influx

The androgen receptor (AR), a member of the subfamily of steroid receptors, has a pivotal role in the regulation of prostate growth and secretory responses. After binding of its ligand, the AR undergoes a conformational change and acquires an active form which regulates transcription of androgen-responsive genes (Kallio et al, 1994). Thereby its functional activity is modulated by interactions with receptor-associated tissue-specific co-regulatory proteins, called co-activators and co-repressors (Ikonen et al, 1994; Yeh and Chang, 1996; Froesch et al, 1998). AR mRNA and protein in the prostate are differentially regulated by androgens; androgen treatment leads to down-regulation of AR mRNA but stabilizes the protein (Krongrad et al, 1991; Zhou et al, 1995). In consequence, stabilization of AR protein results in a net increase in AR expression. The regulation of mRNA for the AR is, however, cell-type specific. In contrast to prostatic tissue, AR mRNA in bone cells is up-regulated by androgen (Wiren et al, 1997). In addition to androgenic hormones, some polypeptide growth factors, cytokines and compounds that increase intracellular cAMP levels participate in the regulation of AR expression. Epidermal growth factor (EGF) decreases the levels of AR mRNA as well as AR protein in the human prostatic carcinoma cell line LNCaP (Mizokami et al, 1992; Henttu and Vihko, 1993). Moreover, it was shown that interleukin-1β (IL-1β), which is present in conditioned medium from peripheral blood monocytes, is responsible for down-regulation of AR protein expression (Culig et al, 1998). AR protein levels were also found to be reduced in response to retinoic acid (Young et al, 1994). In contrast, cAMP increases AR expression at the transcriptional level and cAMP response element was described in the human AR gene promoter (Mizokami et al, 1994).

Until now, effects of basic fibroblast growth factor (bFGF) on AR protein expression in prostate cancer have not been studied. bFGF is a multifunctional peptide that is the prototype of heparin-binding growth factors (Bikfalvi, 1995). Studies from our laboratory and others showed higher bFGF serum levels in patients with carcinoma of the prostate compared with healthy controls (Meyer et al, 1995; Cronauer et al, 1997*a*). In some cases, prostate tumour progression is associated with an increase in serum bFGF (Cronauer et al, 1997*a*). bFGF itself is expressed in prostate cancer cells and in tissue specimens (Nakamoto et al, 1992; Cronauer et al, 1997*a*). AR expression was unequivocally demonstrated in all histological types of prostate cancer and in metastatic lesions (Van der Kwast et al, 1991; Hobisch et al, 1995, 1996). Previous work revealed negative effects of bFGF on AR-regulated prostate-specific antigen (PSA) transcripts in LNCaP cells (Gleave et al, 1992). Based on that study, we asked whether bFGF effects on PSA may be due to down-regulation of expression of the AR. LNCaP cells were derived from a lymph node metastasis from human carcinoma of the prostate and express high amounts of the mutated AR, that is activated by various steroid hormones and

*Received 9 February 1999*
*Revised 21 June 1999*
*Accepted 22 June 1999*

*Correspondence to:* Z Culig

*Correspondence to:* MV Cronauer, Department of Urology, Northwestern University Medical School, Robert H Lurie Comprehensive Cancer Center – Olson 8360, 710 N Fairbanks Court, Chicago, IL 60611, USA

non-steroidal anti-androgens (Veldscholte et al, 1990). In the present report, we have investigated the regulation of AR protein and mRNA expression by bFGF in LNCaP cells.

### MATERIALS AND METHODS

### Materials

The human prostate tumour cell line LNCaP was purchased from the American Type Culture Collection (Rockville, MD, USA). MCDB-131 medium, dihydrotestosterone (DHT) and calcium ionophore A 23187 were from Sigma (St Louis, MO, USA). Fetal calf serum (FCS) was purchased from Biological Industries (Kibbutz Beth Haemek, Israel). Trypsin–EDTA was from HyClone (Cramlington, UK). Phosphate-buffered saline (PBS) was obtained from PAA Laboratories (Linz, Austria). Cell culture plasticware was from Falcon (Lincoln, NE, USA), Costar (Cambridge, MA, USA) and Sarstedt (Nürmbrecht, Germany). bFGF was purchased from Biomol (Hamburg, Germany). AR-specific rabbit antiserum PG-21 was from Paesel-Lorei (Frankfurt/M, Germany) and peroxidase-labelled mouse anti-rabbit immunoglobulin was a product of Santa Cruz Biotechnologies (Santa Cruz, CA, USA). Western blotting detection reagents were from Amersham (Little Chalfont, UK). Unlabelled and $^3$H-labelled methyltrienolone (R1881) were purchased from New England Nuclear (Dreieichenhain, Germany). Fura-2 was obtained from Molecular Probes (Eugene, OR, USA).

### Cell culture

LNCaP cells were grown at 37°C in a humidified atmosphere of 5% carbon dioxide in air. The cells were routinely cultured in MCDB-131 supplemented with 5% FCS, penicillin (120 μg ml$^{-1}$) and streptomycin (120 U ml$^{-1}$). In all experiments, 5% charcoal-stripped (CS) FCS was used.

### Determination of prostate-specific antigen in the supernatants of LNCaP cells

LNCaP cells were seeded into 24-well plates and allowed to adhere for 24 h. Then they were cultured for 24, 48 and 72 h respectively, with DHT in the absence or presence of increasing concentrations of bFGF. Afterward, the medium was removed and PSA was assessed by a microparticle enzyme immunoassay IMx (Abbott Laboratories, Abbott Park, IL, USA). The sensitivity of the assay was calculated to be 0.02 ng ml$^{-1}$. PSA levels were normalized according to cell numbers which were determined using a coulter counter (Coulter Electronics, Harpenden, Herts, UK).

### Immunoblotting of the androgen receptor

LNCaP cells were plated in 6-well culture dishes and cultured in the absence or presence of bFGF. DHT was added into culture media because (i) the experiments were carried out under the same conditions as PSA measurements and (ii) previous reports demonstrated positive effect of DHT on AR stabilization (Krongrad et al, 1991; Zhou et al, 1995). Cell monolayers were detached by trypsinization. Cell pellets were harvested by centrifugation in a tabletop centrifuge (3 min, 1500 ***g***). They were resuspended in a phosphate buffer (pH 7.5, 20 mM $NaH_2PO_4$, 1 mM EDTA, 10% glycerol and 0.1% monothioglycerol) and homogenized with an ultrasound disintegrator Sonifier 250 (Branson, Danburry, CT, USA). Protein concentration in homogenates was measured by the method of Bradford (1978). Equal amounts of protein from each sample were separated by sodium dodecyl sulphate-polyacrylamide gel electrophoresis on a 8%-gel and transferred to the membranes. Non-specific binding was blocked by incubating the membrane with 5% non-fat dry milk in PBS. Afterward, the membrane was exposed to the AR-specific antiserum PG-21 (dilution 1:500) for 2 h at room temperature. After 3 washing cycles, the immunocomplex was detected by incubating the membrane with peroxidase-labelled mouse anti-rabbit immunoglobulin for another 2 h. AR bands were visualized by enhanced chemiluminescence.

### AR binding studies

After incubation with either bFGF or the calcium ionophore A 23187, LNCaP cells were harvested, washed once and resuspended in MCDB-131 supplemented with 5% CS FCS. In order to determine specific binding of the non-metabolizable androgen R1881, the cells were subsequently incubated with increasing concentrations of $^3$H-labelled R1881. Non-specific binding was measured in the presence of a 200-fold molar excess of unlabelled R1881. After 90 min incubation, cell pellets were recovered by centrifugation (3 min, 3800 ***g***) and washed twice in ice-cold medium. Afterward, cell pellets were lysed in a scintillation liquid Optiphase and radioactivity was determined in a β-scintillation counter Wallac 1410 (Pharmacia, Turku, Finland). $B_{max}$ and $K_d$ were calculated by means of Scatchard analysis.

### Measurement of intracellular calcium concentrations

Calcium release from intracellular stores and store-operated calcium influx after treatment with the calcium ionophore A 23187 or bFGF were measured by single cell fluorescence technique employing fura-2 as previously described (Grynkiewicz et al, 1985; Tinhofer et al, 1996). Cells were grown on sterile slide covers for 24 h and labelled with 1 μM fura-2 for 30 min. Afterward, the cells were washed with HBS (140 mM sodium chloride (NaCl), 5 mM potassium chloride (KCl), 1 mM calcium chloride ($CaCl_2$), 0.5 mM magnesium chloride ($MgCl_2$), 5.5 mM glucose) for at least 30 min at room temperature. For determination of the cytoplasmic calcium concentration, the slide was placed into the recording chamber and the cells were kept further in HBS. The intracellular calcium concentration after treatment was calculated from the ratios of background subtracted images (excitation wavelength 340 and 380 nm, emission wavelength 510 nm) according to the calibration procedure described elsewhere (Grynkiewicz et al, 1985).

### Determination of androgen receptor mRNA levels by semiquantitative RT-PCR

Cell pellets were obtained after incubation of LNCaP cells with serum only, DHT, bFGF and a combination thereof respectively. Total RNA was extracted from cell pellets using a guanidinium thiocyanate–acid phenol–chlorophorm (pH 4.0) extraction technique (Eder et al, 1997). Reverse transcription was performed with 500 ng of RNA in 40 μl containing finnzyme buffer [20 mM

 © 2000 Cancer Research Campaign

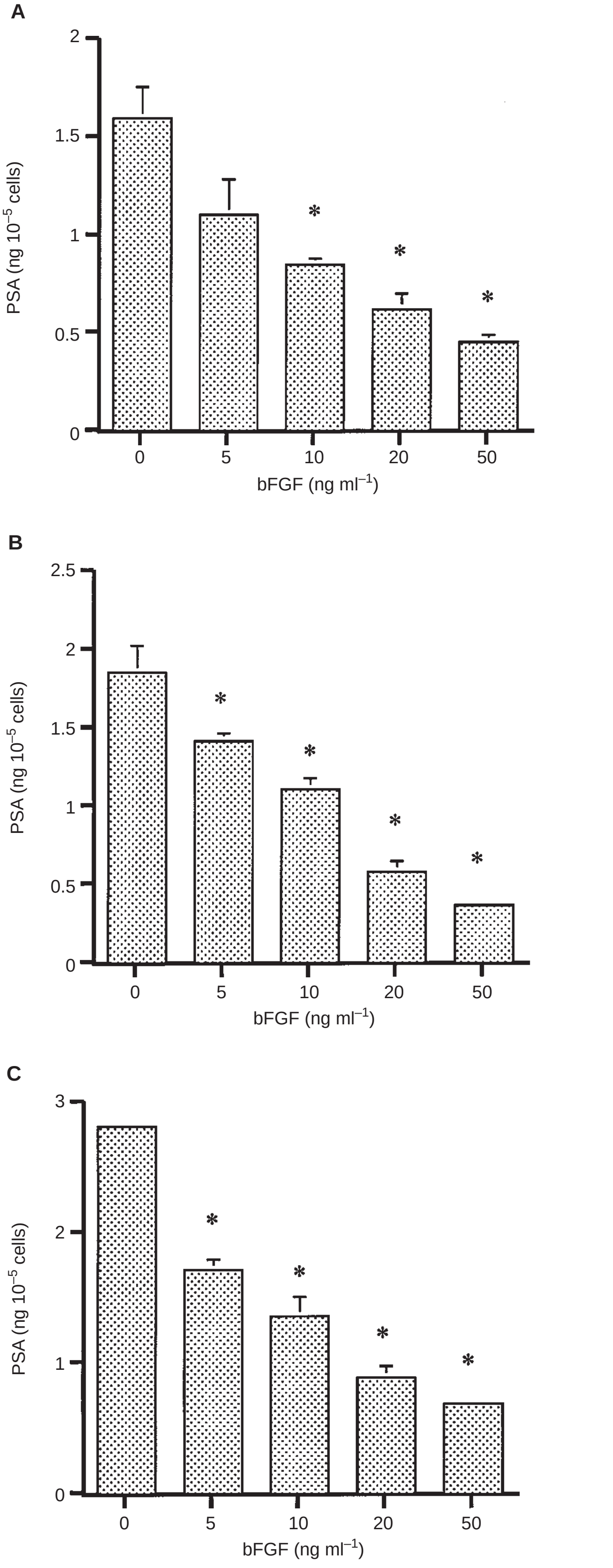

**Figure 1** Down-regulation of PSA protein secretion in LNCaP cells by bFGF. The cells were incubated in the presence of 10 nM of DHT with increasing concentrations of bFGF for (**A**) 24, (**B**) 48 and (**C**) 72 h. PSA levels in the supernatants were measured by an enzyme immunoassay and normalized according to cell numbers. Mean values were calculated from three experiments; bars, s.e.m.; **P* < 0.05, bFGF treatment vs untreated control, Mann–Whitney *U*-test potassium phosphate, pH 7.2, 0.2 mM dithiothreitol (DTT), 0.2% Triton X-100, 5% glycerol], 0.5 mM dNTPs, 200 pmol $N_6$-primers, 0.15% β-mercaptoethanol, 0.1 mg ml$^{-1}$ bovine serum albumin, 39 U ribonuclease inhibitor and 10 U finnzyme AMV reverse transcriptase in a thermocycler. Thermocycler settings were as follows: 8 min at 20°C, 8 min at 25°C and 30 min at 42°C (4 cycles). Polymerase chain reaction (PCR) was performed with 2 µl of cDNA solution in a final volume of 50 µl PCR reaction buffer (10 mM Tris–HCl pH 8.8, 1.5 mM $MgCl_2$, 50 mM KCl, 0.1% Triton X-100) containing 0.1 mM of each dNTP, 1.25 U of Dynazyme polymerase and 12.5 pmol of each primer. An AR cDNA fragment and a cDNA fragment of the housekeeping gene β2-microglobulin were amplified in a duplex PCR. Amplification was started with AR primers alone for 4 cycles. Thereafter, β2-microglobulin primers were added and the amplification was continued for another 20 cycles. The thermocycler programme used was as follows: a precycle of 3 min at 95°C followed by 24 cycles of 25 s at 94°C, 10 s at 96°C, 1 min at 57°C, 30 s at 73°C and a postcycle of 2 min at 73°C. The primers used for AR detection were AR 2047/20 sense 5′-ATG GTG AGC AGA GTG CCC TA-3′ (fluorescence-labelled) and 2474/21 antisense 5′-GTG GTG CTG GAA GCC TCT CCT-3′. The primers used for the amplification of β2-microglobulin were sense 5′-ATG CCT GCC GTG TGA ACC ATG T-3′ and antisense 5′-AGA GCT ACC TGT GGA GCA ACC T-3′ (fluorescence-labelled). After PCR amplification, the samples were diluted in formamide, the fragments were separated on a polyacrylamide sequencing gel on an ABI 370A DNA sequencer (ABI, Foster City, CA, USA) and the fluorescence intensities of both fragments were measured using the ABI GeneScan Software. Results are expressed as an AR/β2-microglobulin ratio.

### Statistical analysis

Probability values were calculated by Mann–Whitney *U*-test. *P*-values < 0.05 were considered statistically significant.

### RESULTS

### Effects of bFGF on PSA protein in LNCaP cells

PSA was determined in supernatants from LNCaP cells which were cultured with bFGF in the presence of DHT (Figure 1 A–C). bFGF down-regulated PSA protein in LNCaP cells in a dose-dependent manner, consistent with previous observations made by Gleave and associates (Gleave et al, 1992). This down-regulation was observed after 24, 48 and 72 h of treatment respectively (Figure 1 A–C). With 50 ng ml$^{-1}$ bFGF, an 80% inhibition in PSA protein expression was observed (Figure 1B). Based on these findings, we investigated whether the inhibition of PSA protein is preceded by negative effects of bFGF on AR expression.

### Regulation of AR protein expression by bFGF

AR protein expression in LNCaP cells after treatment with bFGF was studied by Western blotting and a radioligand binding assay. When cells were grown in the presence of DHT AR was down-regulated by bFGF, as revealed by Western blotting (Figure 2). bFGF action was concentration-dependent. The lowest AR level was observed after treatment with 50 ng ml$^{-1}$ of bFGF. This finding was confirmed by measurement of specific binding of

*© 2000 Cancer Research Campaign*

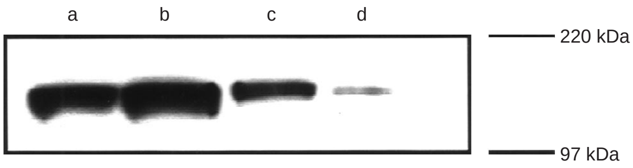

**Figure 2** Immunoblot analysis of AR protein expression. LNCaP cells were cultured for 72 h in the absence (lane a) or presence of DHT (lanes b–d) with bFGF at concentrations of 25 (lane c) or 50 ng $ml^{-1}$ (lane d). Equal amounts of protein from each sample were separated by sodium dodecyl sulphate-polyacrylamide gel electrophoresis. Immunodetection was performed with the anti-AR antibody PG-21 as described in Materials and Methods. This figure is a representative of three experiments

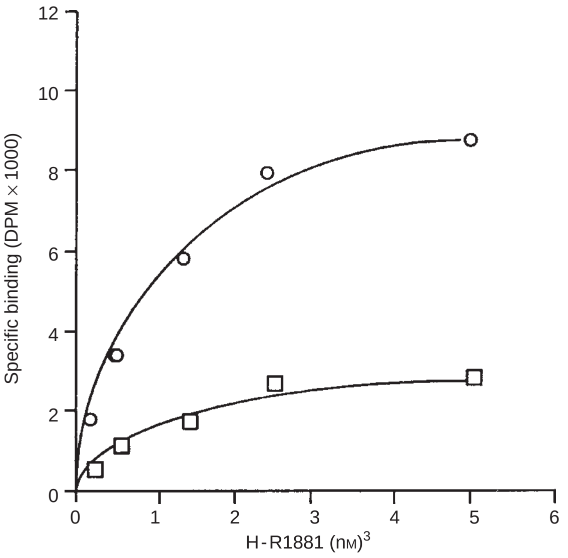

**Figure 3** Specific androgen binding in bFGF-treated LNCaP cells. LNCaP cells were cultured in the absence (control) or presence of bFGF (10 ng $ml^{-1}$) in the absence of DHT for 24 h. Subsequently, the cells were scraped off and incubated for 90 min at room temperature with increasing concentrations of $^3$H-R1881. Nonspecific binding was measured in the presence of a 200-fold molar excess of unlabelled R1881. After incubation, the cells were washed twice in ice-cold medium and specific binding was determined. $B_{max}$ and $K_d$ were calculated by Scatchard analysis. (○) Control, $K_d$ = 1.4 nmol, $B_{max}$ = 0.80 fmol $\mu g^{-1}$ protein; (□) 10 ng $ml^{-1}$ bFGF, $K_d$ = 1.8 nmol, $B_{max}$ = 0.25 fmol $\mu g^{-1}$ protein

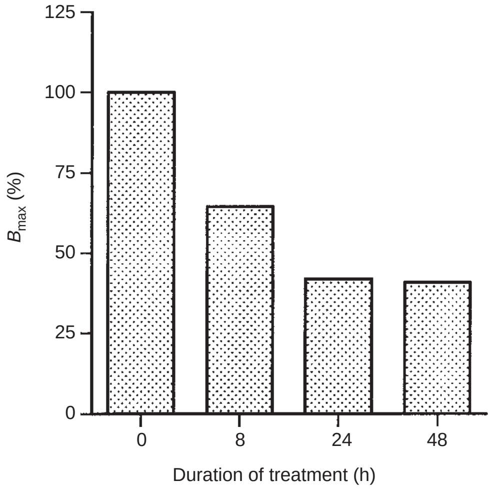

**Figure 4** Time course of bFGF effect on AR expression. LNCaP cells were treated with 10 ng $ml^{-1}$ bFGF in the absence of DHT for 8, 24 and 48 h and specific binding was determined as described in the legend for Figure 3. Two experiments were performed. $B_{max}$ at each time point is expressed as percent of $B_{max}$ measured in untreated cells

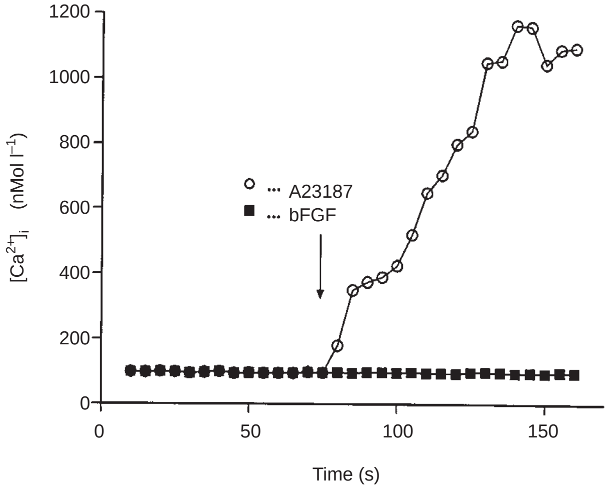

**Figure 5** Measurement of intracellular calcium concentrations by single cell fluorescence technique in LNCaP cells. The cells were grown on sterile slide covers and labelled with fura-2. They were treated with either calcium ionophore A 23187 or bFGF in the absence of DHT. The measurement was performed as described elsewhere (Grynkiewicz et al, 1985)

$^3$H-labelled R1881 (data not shown). Binding studies showed also a down-regulation of AR when cells were treated with bFGF in the absence of DHT (Figure 3). Taken together, these results indicate that down-regulation of AR by bFGF does not depend on the presence of DHT. Subsequently, we analysed time-course of the bFGF effect by measurement of androgen binding after treatment with bFGF for 8, 24 and 48 h (Figure 4). AR protein expression decreased already after 8 h of treatment and a maximal inhibition was observed 24 h after addition of bFGF to culture media.

### Analysis of calcium release induced by bFGF

In LNCaP cells, an elevation of intracellular calcium levels was shown to be associated with a decrease in AR mRNA and protein (Gong et al, 1995). The ability of the calcium ionophore A 23187 to down-regulate AR expression was confirmed in our experiments. AR protein level was reduced by 40% after 14 h treatment with 1 µM of A 23187 (data not shown). bFGF is known to increase calcium influx in several cell types (Cheng et al, 1993; Merle et al, 1997). Therefore, the possibility that bFGF action on AR expression is mediated by an elevation of intracellular calcium levels was examined. As shown in Figure 5, A 23187 was able to induce calcium influx and an increase in cytoplasmic calcium concentration in LNCaP cells whereas bFGF was incapable of eliciting a similar effect. We concluded that bFGF does not regulate AR protein by modulating intracellular calcium levels.

### Analysis of AR mRNA levels

In order to investigate how bFGF affects AR mRNA, LNCaP cells were treated with bFGF for 24 h and AR mRNA was determined

© 2000 Cancer Research Campaign

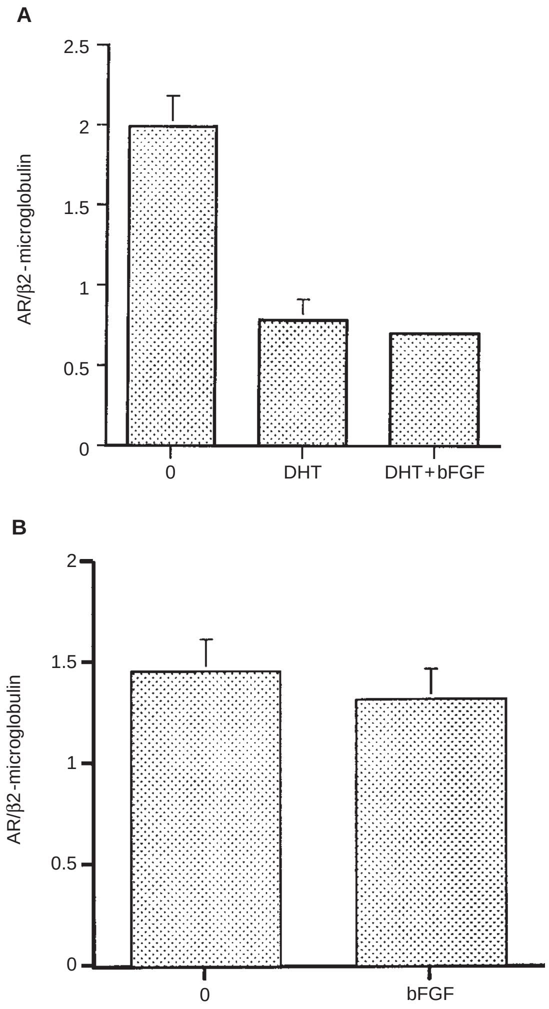

**Figure 6** Measurement of AR mRNA levels in LNCaP cells by semiquantitative RT-PCR. AR mRNA levels were determined after treatment with (**A**) DHT (10 nM) or DHT and bFGF (50 ng ml$^{-1}$) and (**B**) bFGF for 24 h. mRNA levels are expressed as an AR/β2-microglobulin ratio. Three independent experiments were performed and mean values were calculated; bars, s.e.m.

by semiquantitative reverse transcription PCR (RT-PCR). Treatment with DHT alone led to the down-regulation of AR mRNA by 61% compared to untreated cells (Figure 6A). After treatment with DHT and bFGF, AR mRNA did not decrease further. The mean AR/β2-microglobulin ratio in cells treated with DHT and bFGF was $0.7 \pm 0.03$, whereas the cells incubated with DHT only had an AR/β2-microglobulin ratio of $0.78 \pm 0.13$. The measurement of AR mRNA was also performed in LNCaP cells treated with bFGF in the absence of DHT. There was no difference between AR mRNA levels in cells treated with bFGF compared to untreated controls (Figure 6B). Taken together, the results of these experiments show that bFGF does not affect steady-state levels of AR mRNA.

### DISCUSSION

In the present study, we show that AR protein in human prostate cancer cells is down-regulated by bFGF. This down-regulation precedes the decrease in PSA protein measured in LNCaP supernatants. Negative regulation of AR expression by a growth factor was previously reported for EGF (Mizokami et al, 1992; Henttu and Vihko, 1993). It seems, however, that bFGF and EGF modulate AR expression by different mechanisms because bFGF, in contrast to EGF, does not diminish AR mRNA levels. We hypothesize that bFGF action is exerted through degradation of the receptor protein. An opposite effect, stabilization of AR protein by androgen was previously demonstrated by Zhou and associates (Zhou et al, 1995). Our observation that bFGF does not reduce AR mRNA levels is supported by a recent paper by Russell and associates (1999). Those authors did not observe differences in AR mRNA levels between LNCaP cells stably transfected with the bFGF expression vector and the parental LNCaP cells. Interestingly, bFGF-transfected cells showed a reduced growth rate in response to androgen. This may be due to down-regulation of AR protein by bFGF.

An elevation of intracellular calcium is implicated in down-regulation of the AR in LNCaP cells (Gong et al, 1995). bFGF is known to increase intracellular calcium levels in various cell systems (Cheng et al, 1993; Merle et al, 1997). Therefore, we initially hypothesized that this mechanism is operative in prostate cancer cells. To verify this hypothesis, we applied an established single cell fluorescence method to determine calcium levels. However, we did not measure an increase in intracellular calcium levels following bFGF treatment. Interestingly, Wasilenko and associates recently reported that neither of the calcium agonists tested was able to induce an elevation of intracellular calcium in LNCaP cells (Wasilenko et al, 1997). Thus, other signalling pathways must be considered for inhibition of AR expression by bFGF. bFGF regulates cellular growth and differentiation by involving protein kinase C (Hrzenjak and Shain, 1997), mitogen-activated protein kinase (Hurley et al, 1996; Milasincic et al, 1996), phosphatidylinositol 3-kinase (Raffioni and Bradshaw, 1992) or phospholipase signalling (Sa and Fox, 1994; Suzuki et al, 1996).

bFGF was identified as a major autocrine growth factor produced by prostate fibroblasts (Story et al, 1989). In later studies, it was demonstrated that prostate epithelial cells are also a source of bFGF (Nakamoto et al, 1992; Sherwood et al, 1992; Cronauer et al, 1997*a*). The receptors for bFGF are expressed in both mesenchymal and epithelial prostate cells (Nakamoto et al, 1992; Sherwood et al, 1992). Some observations from in vitro studies support the view that down-regulation of the AR by bFGF may be relevant to prostate cancer model systems. In the bFGF-producing prostate cancer cell lines PC-3 and DU-145 as well as in prostatic primary tissue cultures the AR is down-regulated (Tilley et al, 1990; Grant et al, 1996). Nearly all media for primary prostate cultures contain bovine pituitary extract that is a rich source of bFGF (Chaproniere and McKeehan, 1986; Peehl and Stamey, 1986; Baird and Böhlen, 1991; Cronauer et al, 1997*b*). This fact may provide a partial explanation for an absent or very low expression of the AR in primary cultures of prostatic epithelial cells. Conversely, androgen-sensitive LNCaP cells did not express bFGF (Cronauer et al, 1997*a*).

Data on bFGF expression in prostate cancer clinical specimens are scarce. However, we reported that in a limited number of specimens an increased bFGF expression was associated with a more advanced tumour stage (Cronauer et al, 1997*a*). Immunohistochemical studies utilizing double staining for bFGF and the AR in prostate tumour specimens might be helpful for understanding implications of AR regulation by bFGF in vivo.

*© 2000 Cancer Research Campaign*

It became clear that the AR is expressed in all prostate tumours even in those that do not respond to endocrine therapy and that currently available anti-androgens may enhance AR activity in advanced carcinoma of the prostate (Van der Kwast et al, 1991; Hobisch et al, 1995, 1996; Peterziel et al, 1995). Identification of signalling cascades which are responsible for AR down-regulation by bFGF might therefore be useful for development of new approaches to control advanced carcinoma of the prostate by modulation of AR levels.

In contrast to IL-1β and retinoic acid that down-regulate the AR and inhibit cellular proliferation, bFGF stimulates the proliferation of LNCaP cells (Nakamoto et al, 1992; Young et al, 1994; Cronauer et al, 1997*a*; Culig et al, 1998). Application of bFGF antisense oligonucleotides in rat prostate tumours led to inhibition of cell growth (Shain et al, 1996). Taken together, these findings indicate that AR content is only one determinant of mitogenic potential of prostate carcinoma cells.

In summary, our studies show that bFGF is a potent negative regulator of AR protein level in the human prostate cancer cell line LNCaP. The underlying molecular mechanisms remain to be elucidated in future studies.

### ACKNOWLEDGEMENTS

This work was supported by Austrian Science Foundation (FWF SFB 002-F 203) and Ministère de I'Education Nationale et de la Formation Professionelle Luxembourg. The authors thank Dr Lynn Janulis for helpful suggestions and Kukka Strese for help in measurement of intracellular calcium concentrations. The authors gratefully acknowledge the expert technical assistance of T Sierek, U Plawenn, M Pöschl, G Hölzl and P Dertschnig and editorial assistance of R Schober.

### REFERENCES

Baird A and Böhlen P (1991) Fibroblast growth factors. In: *Peptide Growth Factors and Their Receptors I*, Sporn MB and Roberts AB (eds), pp. 369–418. Springer: Heidelberg

Bikfalvi A (1995) Significance of angiogenesis in tumour progression and metastasis. *Eur J Cancer* **31A**: 1101–1104

Bradford M (1978) A rapid and sensitive method for the quantitation of microgram quantities of protein using the principle of protein-dye binding. *Anal Biochem* **72**: 248–254

Chaproniere DM and McKeehan WL (1986) Serial culture of single adult human prostatic epithelial cells in serum-free medium containing low calcium and a new growth factor from bovine brain. *Cancer Res* **46**: 819–824

Cheng B, McMahon DG and Mattson MP (1993) Modulation of calcium current, intracellular calcium levels and cell survival by glucose deprivation and growth factors in hippocampal neurons. *Brain Res* **607**: 275–285

Cronauer MV, Hittmair A, Eder IE, Hobisch A, Culig Z, Ramoner R, Zhang J, Bartsch, G, Reissigl A, Radmayr C, Thurnher M and Klocker H (1997*a*) Basic fibroblast growth factor levels in cancer cells and in sera of patients suffering from proliferative disorders of the prostate. *Prostate* **31**: 223–233

Cronauer MV, Eder IE, Hittmair A, Sierek G, Hobisch A, Culig Z, Thurnher M, Bartsch G and Klocker H (1997*b*) A reliable system for the culture of human prostatic cells. *In Vitro Cell Dev Biol Anim* **33**: 742–744

Culig Z, Hobisch A, Herold M, Hittmair A, Thurnher M, Eder IE, Cronauer MV, Rieser C, Ramoner R, Bartsch G, Klocker H and Konwalinka G (1998) Interleukin-1β mediates the modulatory effects of monocytes on LNCaP human prostate cancer cells. *Br J Cancer* **78**: 1004–1011

Eder IE, Stenzl A, Hobisch A, Cronauer MV, Bartsch G and Klocker H (1997) Expression of transforming growth factors beta-1, beta 2 and beta 3 in human bladder carcinomas. *Br J Cancer* **75**: 1753–1760

Froesch BA, Takayama S and Reed JC (1998) BAG-1L protein enhances androgen receptor function. *J Biol Chem* **273**: 11660–11666

Gleave ME, Hsieh JT, Wu HC, von Eschenbach AC and Chung LWK (1992) Serum prostate specific antigen levels in mice bearing human prostate LNCaP tumors are determined by tumor volume and endocrine and growth factors. *Cancer Res* **52**: 1598–1605

Gong Y, Blok LJ, Perry JE, Lindzey JK and Tindall DJ (1995) Calcium regulation of androgen receptor expression in the human prostate cancer cell line LNCaP. *Endocrinology* **136**: 2172–2178

Grant ES, Batchelor KW and Habib FK (1996) Androgen independence of primary epithelial cultures of the prostate is associated with a down-regulation of androgen receptor gene expression. *Prostate* **29**: 339–349

Grynkiewicz G, Poenie M and Tsien RY (1985) A new generation of $Ca^{2+}$ indicators with greatly improved fluorescence properties. *J Biol Chem* **260**: 3440–3450

Henttu P and Vihko P (1993) Growth factor regulation of gene expression in the human prostatic carcinoma cell line LNCaP. *Cancer Res* **53**: 1051–1058

Hobisch A, Culig Z, Radmayr C, Bartsch G, Klocker H and Hittmair A (1995) Distant metastases from prostatic carcinoma express androgen receptor protein. *Cancer Res* **55**: 3068–3072

Hobisch A, Culig Z, Radmayr C, Bartsch G, Klocker H and Hittmair A (1996) Androgen receptor status of lymph node metastases from prostate cancer. *Prostate* **28**: 129–135

Hrzenjak M and Shain SA (1997) Fibroblast growth factor-2 and TPA enhance prostate cancer cell proliferation and activate members of the ras and PKC signal transduction pathways. *Recept Signal Tranduct* **7**: 207–219

Hurley MM, Marcello K, Abreu C and Kessler M (1996) Signal transduction by basic fibroblast growth factor in rat osteoblastic Py1a cells. *J Bone Mineral Res* **11**: 1256–1263

Ikonen T, Palvimo JJ, Kallio PJ, Reinikainen P and Jänne OA (1994) Stimulation of androgen-regulated transactivation by modulators of protein phosphorylation. *Endocrinology* **135**: 1359–1366

Kallio PJ, Jänne OA and Palvimo JJ (1994) Agonists, but not antagonists, alter the conformation of the hormone-binding domain of androgen receptor. *Endocrinology* **134**: 998–1001

Krongrad A, Wilson CM, Wilson JD, Allman DR and McPhaul MJ (1991) Androgen increases androgen receptor protein while decreasing receptor mRNA in LNCaP cells. *Mol Cell Endocrinol* **76**: 79–88

Merle PL, Usson Y, Robert-Nicoud M and Verdetti J (1997) Basic FGF enhances calcium permeable channel openings in adult rat cardiac myocytes: implication in the bFGF-induced increase of free $Ca^{2+}$ content. *J Mol Cell Cardiol* **29**: 2687–2698

Meyer GE, Yu E, Siegal JA, Petteway JC, Blumenstein BA and Brawer MK (1995) Serum basic fibroblast growth factor in men with and without prostate carcinoma. *Cancer* **76**: 2304–2311

Milasincic DJ, Calera MR, Farmer SR and Pilch PF (1996) Stimulation of C2C12 myoblast growth by basic fibroblast growth factor and insulin-like growth factor 1 occur via mitogen-activated protein kinase-dependent and -independent pathways. *Mol Cell Biol* **16**: 5964–5973

Mizokami A, Saiga H, Matsui T, Mita T and Sugita A (1992) Regulation of androgen receptor by androgen and epidermal growth factor in a human prostatic cancer cell line, LNCaP. *Endocrinol Jpn* **39**: 235–243

Mizokami A, Yeh SY and Chang C (1994) Identification of 3′5′-cyclic adenosine monophosphate response element and other cis-acting elements in the human androgen receptor gene promoter. *Mol Endocrinol* **8**: 77–88

Nakamoto T, Chang CS, Li AK and Chodak GW (1992) Basic fibroblast growth factor in human prostate cancer cells. *Cancer Res* **52**: 571–577

Peehl DM and Stamey TA (1986) Serum-free growth of adult human prostatic epithelial cells. *In Vitro Cell Dev Biol* **22**: 82–90

Peterziel H, Culig Z, Stober J, Hobisch A, Radmayr C, Bartsch G, Klocker H and Cato AC (1995) Mutant androgen receptors in prostatic tumors distinguish between amino-acid-sequence requirements for transactivation and ligand binding. *Int J Cancer* **63**: 544–550

Raffioni S and Bradshaw RA (1992) Activation of phosphatidylinositol 3-kinase by epidermal growth factor, basic fibroblast growth factor, and nerve growth factor in PC12 pheocromocytoma cells. *Proc Natl Acad Sci USA* **89**: 9121–9125

Russell PJ, Bennett S, Joshua A, Yu Y, Downing SR, Hill MA, Kingsley EA, Mason RS and Berry J (1999) Elevated expression of FGF-2 does not cause prostate cancer progression in LNCaP cells. *Prostate* **40**: 1–13

Sa G and Fox PL (1994) Basic fibroblast growth factor-stimulated endothelial cell movement is mediated by a pertussis toxin-sensitive pathway regulating phospholipase A2 activity. *J Biol Chem* **269**: 3219–3225

Shain SA, Saric T, Ke LD, Nannen D and Yoas S (1996) Endogenous fibroblast growth factor-1 or fibroblast growth factor-2 modulate prostate cancer cell proliferation. *Cell Growth Diff* **7**: 573–586

 *© 2000 Cancer Research Campaign*

Sherwood E, Lee C and Kozlowski JM (1992) Basic fibroblast growth factor: a potential mediator of stromal growth in the human prostate. *Endocrinology* **130**: 2955–2963

Story MT, Livingston B, Baeten L, Schwartz SJ, Jacobs SC, Begun FP and Lawson RK (1989) Cultured human prostate-derived fibroblasts produce a factor that stimulates their growth with properties indistinguishable from basic fibroblast growth factor. *Prostate* **15**: 355–365

Suzuki A, Shinoda J, Kanda S, Oiso Y and Kozawa O (1996) Basic fibroblast growth factor stimulates phosphatidylcholine-hydrolyzing phospholipase D in osteoblast-like cells. *J Cell Biochem* **63**: 491–499

Tilley WD, Wilson CM, Marcelli M and McPhaul MJ (1990) Androgen receptor gene expression in human prostate carcinoma cell lines. *Cancer Res* **50**: 5382–5386

Tinhofer I, Maly K, Dietl P, Hochholdinger F, Mayr S, Obermeier A and Grunicke HH (1996) Differential $Ca^{2+}$ signaling induced by activation of the epidermal growth factor and nerve growth factor receptors. *J Biol Chem* **271**: 30505–30509

Van der Kwast TH, Schalken J, Ruizeveld de Winter JA, van Vroonhoven CCJ, Mulder E, Boersma W and Trapman J (1991) Androgen receptors in endocrine-therapy-resistant human prostate cancer. *Int J Cancer* **48**: 189–193

Veldscholte J, Ris-Stalpers C, Kuiper GGJM, Jenster G, Berrevoets C, Claassen E, van Rooij HCJ, Trapman J and Brinkmann AO (1990) A mutation in the ligand binding domain of the androgen receptor of human LNCaP cells affects steroid binding characteristics and response to anti-androgens. *Biochem Biophys Res Commun* **17**: 534–540

Wasilenko WJ, Cooper J, Palad AJ, Somers KD, Blackmore PF, Rhim JS, Wright GLJ and Schellhammer PF (1997) Calcium signaling in prostate cancer cells: evidence for multiple receptors and enhanced sensitivity to bombesin/GRP. *Prostate* **30**: 167–173

Wiren KM, Zhang X, Chang C, Keenan E and Orwoll ES (1997) Transcriptional up-regulation of the human androgen receptor by androgen in bone cells. *Endocrinology* **138**: 2291–2300

Yeh S and Chang C (1996) Cloning and characterization of a specific coactivator, ARA70, for the androgen receptor in human prostate cells. *Proc Natl Acad Sci USA* **93**: 5517–5521

Young C-F, Murtha PE, Andrews PE, Lindzey JK and Tindall DJ (1994) Antagonism of androgen action in prostate tumor cells by retinoic acid. *Prostate* **25**: 39–45

Zhou ZX, Lane MV, Kemppainen JA, French FS and Wilson EM (1995) Specificity of ligand-dependent androgen receptor stabilization: receptor domain interactions influence ligand dissociation and receptor stability. *Mol Endocrinol* **9**: 208–218

*© 2000 Cancer Research Campaign*
